# Six-month stability following extensive alveolar bone augmentation by sausage technique

**DOI:** 10.1186/s40902-023-00384-8

**Published:** 2023-04-23

**Authors:** Kang-min Kim, Soo-young Choi, Jung-Hyun Park, Heon-Young Kim, Sun-Jong Kim, Jin-Woo Kim

**Affiliations:** 1 Department of Oral and Maxillofacial Surgery, Mok-dong Hospital, School of Medicine, Ewha Womans University, Seoul, Korea; 2 Department of Oral and Maxillofacial Surgery, Department of Oral and Maxillofacial Surgery, Seoul Hospital, School of Medicine, Ewha Womans University, Seoul, Korea

**Keywords:** Sausage technique, Bone augmentation, Bone retention rate

## Abstract

**Background:**

Resorption of alveolar bone is a common sequela of tooth loss and presents a clinical problem, especially in the esthetic zone. When ridge resorption occurs, adequate bone augmentation is essential to obtain satisfactory esthetic results. The purpose of this study was to determine the increase and retention rate of bone height or width in patients who received extensive bone augmentation and to analyze factors affecting its prognosis and stability.

**Methods:**

This study was performed on patients who received extensive bone augmentation by sausage technique at the Department of Oral and Maxillofacial Surgery at Ewha Womans University Mok-dong Hospital from January 1, 2018, to February 28, 2022. CBCT images were taken before and 6 months after surgery to compare the amount of increase in bone height or width at the graft site. They were measured using reliable points such as adjacent implants or cephalometric landmarks, inferior alveolar nerve canals as reference points.

**Results:**

A total of 8 patients underwent extensive bone grafting during the given period (mean age was 53.75 years, 2 males and 6 females). Four patients received horizontal augmentation, and 4 received vertical augmentation. When divided by surgical site, 4 patients are in maxilla and 4 in mandible. The average amount of increase in bone width or bone height was 5.38 mm, and the retention rate was about 79.9% after 6 months. The retention rate of horizontal augmentation was 88.8%, which was higher than that of vertical augmentation, which was 74.5%. The maxillary area accounted for 92.2%, and the amount of bone resorption was lower than that of the mandibular area, which was 72.6%. The average stitch out period was about 2.4 weeks, and postoperative dehiscence was observed about 37.5% of the total, more frequently in the mandible (50.0%) than in the maxilla (25.0%).

**Conclusion:**

In conclusion, extensive bone augmentation achieved significant horizontal or vertical bone height or width increase, and the retention rate after 6 months was also high. In addition, surgery in the maxillary region showed a more successful bone augmentation than in the mandible, with a higher maintenance rate and fewer cases of dehiscence.

## Background

Sufficient horizontal alveolar ridge width in proper position is essential to meet the functional and esthetic demands of dental implants [1]. However, sometimes implants cannot be placed in the ideal position due to insufficient alveolar bone width or height. In this case, guided bone regeneration (GBR) should be considered. GBR has been used for horizontal and vertical alveolar bone augmentation and has shown reproducible results with high implant survival rates and low complication rates [2]. Various techniques have been introduced to increase the horizontal width or vertical height of alveolar bone. Among them, it was reported that the sausage technique introduced by Istvan Urban enabled successful bone regeneration by fixing the collagen membrane with a titanium pin and pushing the bone graft material toward the crest [3].

Since the graft material is filled in a sufficient amount inside the fixed membrane, it shows a balloon effect and creates tension in the membrane by pushing the graft material in the crestal direction [1]. Two types of collagen membranes can be used in this technique: cross-linked synthetic collagen membranes or native collagen membranes. Exposure of the cross-linked synthetic collagen membrane can result in loss of approximately 48.5% of the grafted bone [4]. And an inflammatory response may also occur during membrane degradation. On the other hand, rapid degradation of the native collagen membrane results in rapid epithelialization upon exposure, resulting in a relatively low risk of infection [5, 6]. In addition, the native collagen membrane becomes vascularized within the first week of healing, which can reduce the burden on surgeons about patient discomfort and complications [7].

According to the PASS (primary closure, angiogenesis, space maintenance, stability of wound) principles for successful GBR reported by Wang et al., the sausage technique is considered a predictable procedure as it satisfies most of the principles [8]. Wound stability and space maintenance can be improved with a combination of titanium pins, bone grafts, and membranes. The ballooning effect of the membrane can be achieved by sufficient amount of grafting materials with a slow resorption and titanium pin fixation [3]. The purpose of this study was to determine the increase and retention rate of bone height or width in patients who received extensive bone augmentation by sausage technique and to analyze factors affecting its prognosis and stability.

## Methods

This study and access to patients’ records were approved by the Institutional Review Board of the Ewha Medical Center, Seoul, Korea.

### Patients

A total of 8 patients ranging in age from 19 to 68 years (2 males and 6 females, mean age 53.8 ± 15.4 years) received extensive bone augmentation using sausage technique at the Department of Oral and Maxillofacial Surgery at Ewha Womans University Mok-dong Hospital from January 1, 2018, to February 28, 2022. The patients’ electronic medical and dental records including information on age, sex, type of bone augmentation, grafting material and membrane type used in surgery, stitch out period, existence of dehiscence, and increase of postoperative alveolar crest dimension were retrieved.

### Inclusion criteria

Extensive bone augmentation was defined as a level of bone grafting in which supports or fixtures such as bone screws or bone tacks (Osstem, Seoul, Korea) are essential for sufficient vertical and horizontal bone augmentation.

### Surgical procedure

**Fig. 1:**
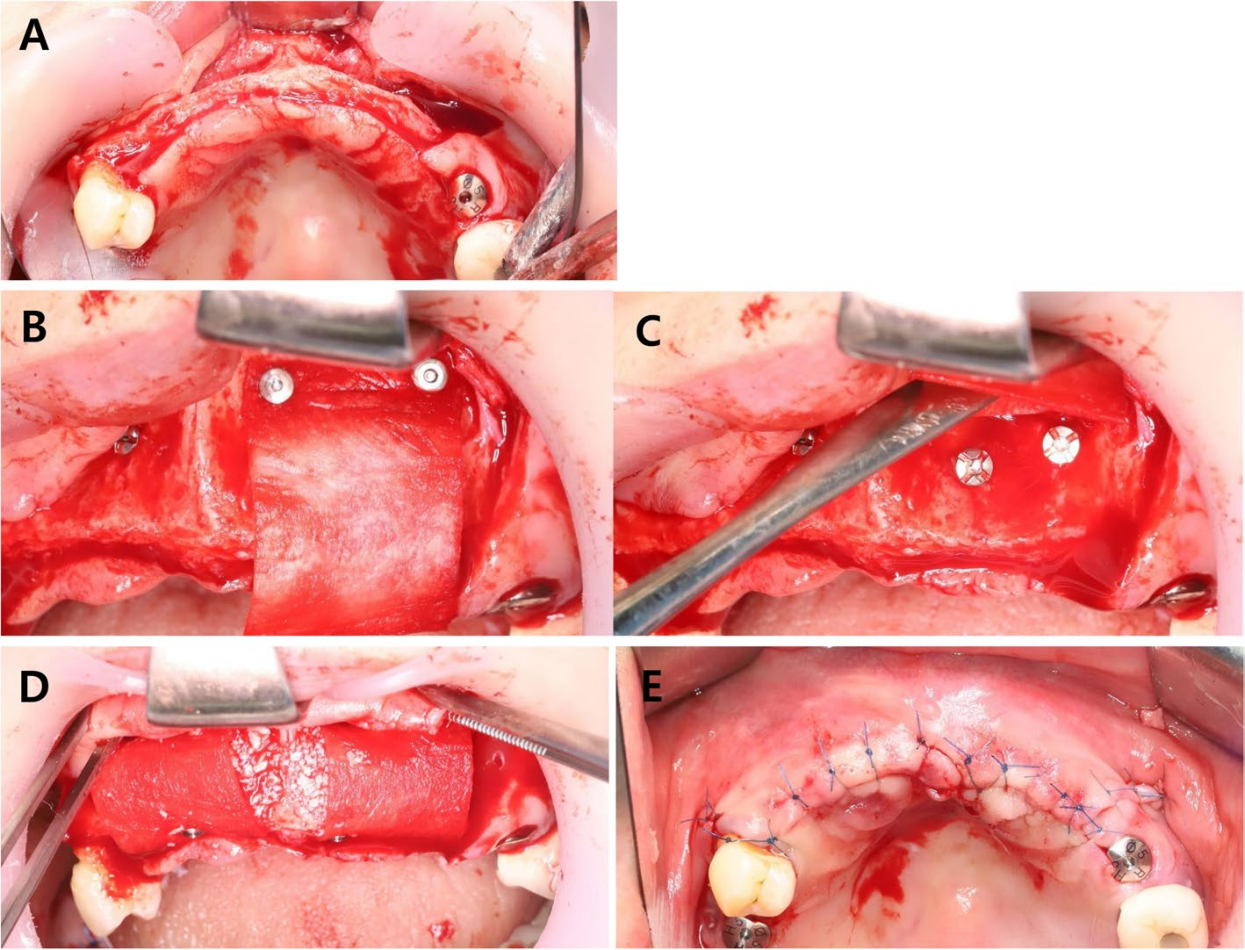
Horizontal bone augmentation using sausage technique in maxillary anterior zone. **A** Incision and flap reflection. **B** and **C** Bone tack and bone screw insertion. **D** and **E** Bone graft and primary closure were done

Before surgery, a preoperative cone-beam computed tomography (CBCT) was taken to assess the amount of residual alveolar bone. The surgical procedure was performed with local anesthesia with the patient under intravenous sedation or general anesthesia, depending on the extent of the lesion and the patient’s general condition. Crestal or vertical incision was done, and flap was reflected on the site to be augmented (Fig. 1A). Bony defect was confirmed. Subsequently, a collagen membrane such as Bio-Gide (Geistlich, Wolhusen, Switzerland) and OssGuide (Osstem, Seoul, Korea) or non-resorbable membrane such as Cytoplast TXT-200 and TI-250 (Osteogenics Biomedical, Lubbock, USA) was fixed to the apical area of the residual alveolar ridge by using bone screws or bone tacks (Osstem, Seoul, Korea) (Fig. 1B). Some screws were fixed in the defect as tenting screws (Fig. 1C). The bone graft materials, an organic bovine bone materials such as Bio-Oss (Geistlich, Wolhusen, Switzerland), A-Oss (Osstem, Seoul, Korea), or allografts harvested from the patient’s iliac crest or alveolar bone, were placed in the defect. The fixed membrane was stretched enough to produce ballooning effect, so it is called sausage technique (Fig. 1D). Primary closure of the mucoperiosteal flap was then achieved (Fig. 1E).

### Measurement of alveolar crest change

**Fig. 2:**
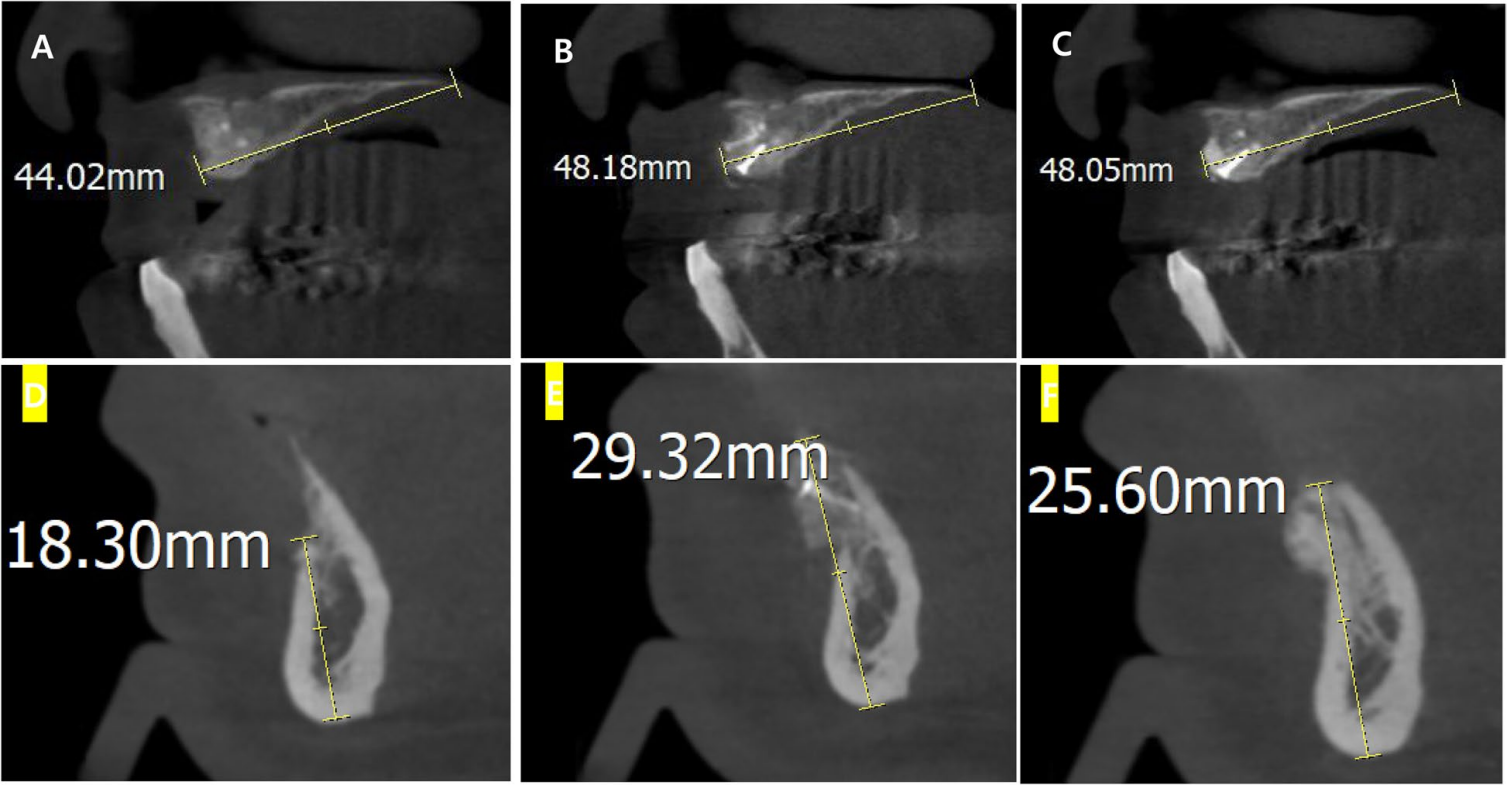
Measuring the amount of increase in bone height or width at the graft site via CBCT. **A** and **D** Preoperative CBCT images. **B** and **E** Postoperative CBCT images right after surgery. **C** and **F** Postoperative CBCT images 6 months after surgery

The postoperative CBCT image was taken three times, preoperatively, right after surgery, and 6 months postoperatively to compare the amount of increase in bone height or width at the graft site. The distance between the bone crest and radiographical reference points in the treatment area was measured such as cephalometric landmarks, inferior alveolar nerve canals, and using PACS (picture archiving and communication system) software (INFINITT PACS 3. 0.9.1, Seoul, Korea) (Fig. 2).

## Results

A total of 8 patients underwent extensive bone grafting during the given period (mean age was 53.8 ± 15.4 years, 2 males and 6 females). The clinical description is shown in Table 1. Four patients received horizontal augmentation, and 4 received vertical augmentation. When divided by surgical site, 4 patients are in maxilla and 4 in mandible. The average amount of increase in bone width or bone height was 5.50 ± 2.41 mm, and the retention rate was about 80.0% after 6 months. The retention rate of horizontal augmentation was 88.8%, which was higher than that of vertical augmentation, which was 74.7%. The maxillary area accounted for 92.2%, and the amount of bone resorption was lower than that of the mandibular area, which was 72.9%. The average stitch out period was about 2.4 weeks. The overall measurement results are displayed in Table 2.

**Table 1:** Clinical case description

Patient	Age, years	Sex	Augmentation (horizontal/vertical)	Grafting material	Membrane
1	60	Female	Vertical	Xenograft	Non-res
2	68	Female	Horizontal	Xenograft	Res
3	59	Female	Vertical	Xenograft	Res
4	48	Female	Horizontal	Allograft	Non-res
5	52	Male	Horizontal	Xenograft/allograft	Non-res
6	63	Female	Horizontal	Xenograft	Res
7	19	Male	Vertical	Xenograft	Res
8	61	Female	Vertical	Xenograft	Res
Stitch out time (weeks)		Dehiscence	Postoperative dimension (mm)	POD (postoperative day) 6-month dimension (mm)	
4		Y	6.5	4.1	
2		N	3.3	3.1	
2		N	4.2	4.0	
1		N	4.6	3.8	
2		Y	4.5	4.1	
2		N	4.4	3.9	
3		N	5.5	4.9	
3		Y	11.0	7.3

**Table 2:** The amount of alveolar bone dimension (mm) and retention rates

Bone augmentation	Postoperative dimension (mm)	POD (postoperative day) 6-month dimension (mm)	Retention rate (%)
Total	5.50 ± 2.41	4.40 ± 1.27	80.00
Maxilla	4.10 ± 0.55	3.78 ± 0.46	92.20
Mandible	6.90 ± 2.84	5.03 ± 1.59	72.90
Vertical	6.80 ± 2.95	5.08 ± 1.54	74.71
Horizontal	4.20 ± 0.61	3.73 ± 0.43	88.81

Extensive alveolar bone augmentation using sausage technique achieved significant horizontal or vertical bone height or width increase, and the retention rate after 6 months was also high. In addition, surgery in the maxillary region showed a more successful bone augmentation than in the mandible, with a higher maintenance rate.

## Discussion

In oral and maxillofacial surgery, vertical or horizontal bone augmentation is often required to ensure adequate bone volume when placing dental implants in edentulous patients, especially in esthetic zones. In recent decades, various surgical techniques for alveolar bone augmentation have been developed and widely practiced by dentists. Byun et al. used tissue expander for vertical augmentation, but there were some limitations that it is difficult to ensure sufficient grafting, difficulty in accurate graft placement, and a decrease in graft stability [9].

In many cases, unsatisfactory results are obtained due to the movement of the grafting materials performing conventional bone augmentation. The augmentation of vertical defects with GBR is limited to 5 mm, resulting in a higher risk of membrane exposure, wound infection, and other complications [10]. Manipulating the graft material in the direction of the crest during the sausage technique would help the aggregation of the graft particles, which leads to better space-maintenance ability [1].

The selection of an appropriate barrier is essential for the success of GBR. The amount of bone augmentation with resorbable membrane was similar to that obtained with the ePTFE, and dehiscence seems to be less frequent when using resorbable membrane compared with non-resorbable ePTFE [11, 12]. And studies conducted by Urban et al. have shown that natural collagen is more elastic than cross-linked synthetic collagen and is a reliable option for anchoring graft materials [2].

Wound stability can be achieved by secure fixation of the membrane, which leads to successful bone regeneration [13]. In this study, it is accomplished by using bone tacks or bone screws and membranes. As a result of the investigation in this study, when using the sausage technique, a significant increase in bone height and width and a high retention rate at 6 months after surgery were confirmed.

There was no failure of bone augmentation due to an inflammatory reaction caused by non-resorbable membrane or titanium pin. Meloni et al. reported a high implant survival rate and bone augmentation without signs of inflammation at the 3-year follow-up without apical pin removal after final loading [14]. Long-term follow-up was not included in this study, but the potential risk seems to be low. Longer follow-up studies are required in the future.

## Conclusion

Within the limitations of this study, the bone augmentation using sausage technique achieved significant horizontal and vertical dimension increase in alveolar bone and also high retention rates after 6 months after surgery.

## Data Availability

Not applicable.
